# Quantitative Analysis of the Physical Properties of Ti6Al4V Powders Used in a Powder Bed Fusion Based on 3D X-ray Computed Tomography Images

**DOI:** 10.3390/ma17040952

**Published:** 2024-02-19

**Authors:** Yuyi Mao, Juan Hu, Qiang Chen, Xiaodong Shen

**Affiliations:** 1 College of Materials Science and Engineering, Nanjing Tech University, Nanjing 211816, China; lwtgzyyx2023@163.com; 2 State Key Laboratory of Materials-Oriented Chemical Engineering, Nanjing Tech University, Nanjing 211816, China; 3 Wuxi Institution of Inspection, Testing and Certification, Wuxi 214028, China

**Keywords:** Ti6Al4V powders, X-ray computed tomography, quantitative characterization, flowability

## Abstract

The physical properties of Ti6Al4V powder affect the spreadability of the powder and uniformity of the powder bed, which had a great impact on the performance of built parts made by powder bed fusion technology. Micro-computed tomography is a well-established technique used to analyze the non-destructivity of the objects’ interior. Ti6Al4V powders were scanned with micro-CT to show the internal and external information of all the particles. The morphology, particle size distribution, hollow particle ratio, density, inclusion, and specific surface area of the powder samples were quantitatively characterized, and the relationship of flowability with these physical properties was analyzed in this work. The research results of this article showed that micro-CT is an effective way to characterize these items, and can be developed as a standard method of powder physical properties in the future.

## 1. Introduction

Powder bed fusion (PBF) is an additive manufacturing process in which thermal energy selectively fuses regions of a powder bed [1]. Laser Powder Bed Fusion (L-PBF), which uses laser as a thermal energy source, is a dominant additive manufacturing technology today and has been widely used in the aerospace, medical, military, etc. [2]. Ti6Al4V powder is a popular commercial feedstock used in L-PBF due to its high strength, low density, high fracture toughness, excellent corrosion resistance, and superior biocompatibility [3]. Its physical properties such as morphology, particle size distribution, flowability, and bulk density have a significant influence on the spreadability of the powder bed and the absorption of laser energy [4,5,6,7], which further affect the quality of the parts produced, including the density, surface finish, and mechanical strength. The commercial spherical powder used in additive manufacturing is mainly produced by atomization methods or plasma spheroidization technology, while the most commonly used technologies to produce Ti6Al4V powders are gas atomization (GA), plasma atomization (PA), and the plasma rotating electrode process (PREP) [8]. There are differences in the physical properties [9] of the powders due to the unique workflow and parameters of the atomization methods or plasma spheroidization process.

ISO TC261 published ISO/ASTM 52907:2019 Additive manufacturing materials to characterize metal powders [10] in 2019. This standard specified the test method for particle size distribution (PSD), chemical composition, characteristic densities, morphology, flowability, and contamination, which had a great impact on the performance of AM parts. Other not mentioned issues in ISO/ASTM 52907:2019, such as the hollow particle ratio and moisture content, also affected the performance of the built parts, and the gases trapped within hollow particles or the moisture on the powder surface can easily transfer into the molten pool during PBF, resulting in gas pores [7]. These methods are used in other industries to characterize the properties of powders. However, it is important to note that these methods may not accurately reflect the state of the powder used in the powder bed fusion process. They are not convenient as every test item has its instrument and these instruments are always located in different labs, which costs more time to finish all the test items. This paper aims to develop a comprehensive method to characterize physical properties based on three-dimensional X-ray computed tomography images.

**Figure 1:**
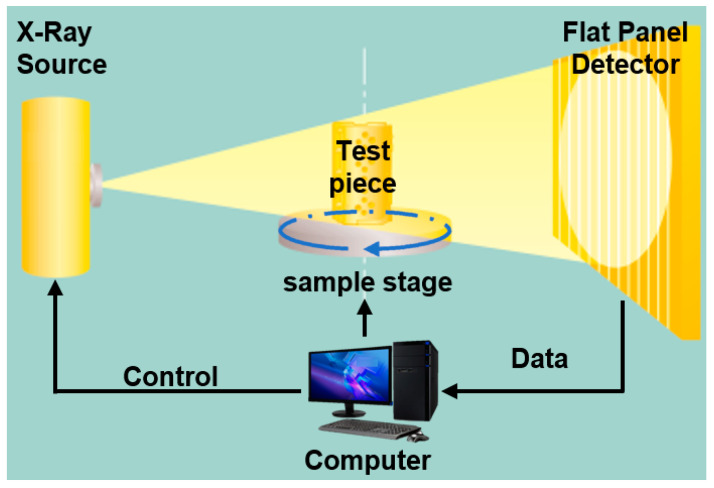
The schematic diagram of X-ray computed tomography equipment.

Micro-computed tomography is a well-established technique used to non-destructively analyze the interior of objects, providing three-dimensional images that carry all the spatial information about a particular powder [11,12,13]. It is used by researchers and companies in the testing of additive manufacturing products to characterize the defects within the parts [14,15,16,17,18], as these parts are generally small in quantity and customized, and some parts are more expensive than machined parts. The schematic diagram of CT is shown in Figure 1. X-ray CT uses a cone beam of X-rays to create a magnified projection image of an object by passing through it. As the X-rays pass through the object, some are absorbed, which reduces the intensity of the X-ray beam that the detector senses. Multiple projection images with different ‘gray values’ are collected by the X-ray detector through object rotation. These images are then reconstructed into 3D voxel data. The material’s radiation absorbability is indicated by the gray value of each voxel. The voxel data can be converted into mesh data or tomographic slice images as needed.

The research results currently available are primarily qualitative analyses, providing 2D or 3D images of powder. This approach is not conducive to analyzing the relationship between powder performance and the quality of the built part. This paper used the 3D images to quantitatively characterize the physical properties of the Ti6Al4V powders and analyze the relationship between the properties tested.

## 2. Material and Experiment

YXLON FF35CT micro-CT (Comet Yxlon, Bern, Switzerland) was used to scan Ti6Al4V powders in this paper and the main parameter is shown in Table 1.

**Figure 2:**
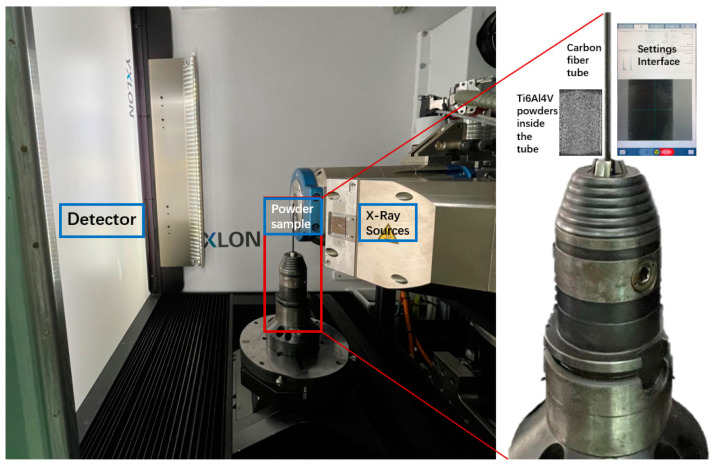
The inside view of FF35CT, a carbon fiber tube powder sample container fixed on the sample stage.

The Ti6Al4V powders used in the PBF process were obtained from 5 different companies and produced through gas atomization (GA). The particle size distribution ranged from 15 to 53 µm. The powder container used in this work was a customized carbon fiber tube with an internal diameter of 1.5 mm. The powder was filled in the container which was then fixed on the scan plate shown in Figure 2. The X-ray source used here was the 190 kV transmission tube, the scan parameters are listed in Table 2, and the scan length of the carbon fiber tube is 1.70 mm. The spatial resolution was adjusted to 1.158 µm accordingly as the particle size distribution was 15–53 µm.

The scan data were then analyzed using Volume Graphics VGStudioMax 2023.2 and Avizo 2023.1.

**Figure 3:**
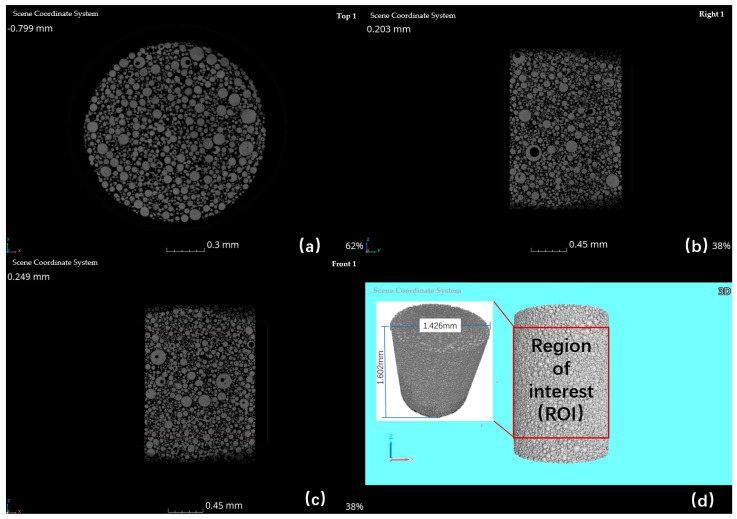
2D CT image of Ti6Al4V powders: (**a**) XY−plane; (**b**) YZ−plane; (**c**) ZX−plane; (**d**) 3D model of the powder sample reconstructed by VGstudioMAX 2023.2, and the region of interest (ROI).

A series of 2D slices of the powder samples were obtained after scanning and these 1800 projections were then reconstructed into a 3D model using VGStudio Max 2023.2 software. A region of interest (ROI) measuring 1.5 mm × 1.5 mm × 1.6 mm was extracted for further analysis as shown in Figure 3.

## 3. CT Image Processing

**Figure 4:**
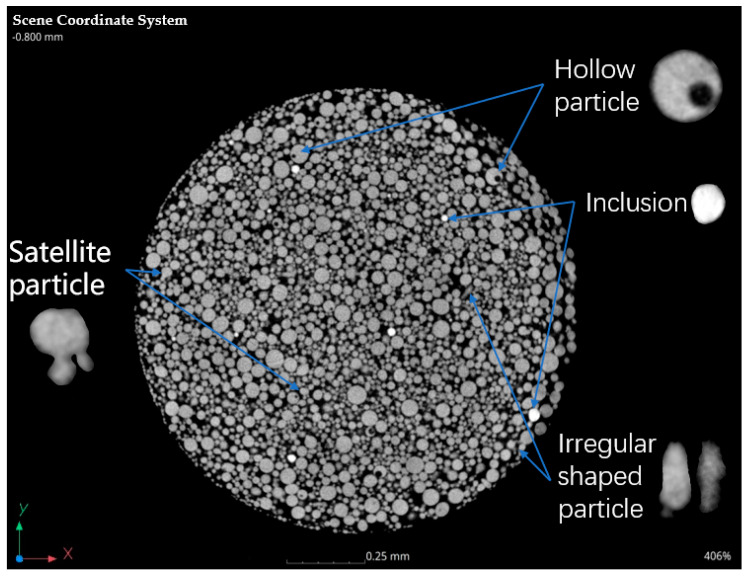
Information shown in the 2D CT image.

The grayscale images of the raw 2D powder slices contained much information about the powder sample such as hollow particles, inclusions, irregularly shaped particles, etc., in the sample, as depicted in Figure 4, which can be used to analyze the physical properties of Ti6Al4V powder.

**Figure 5:**
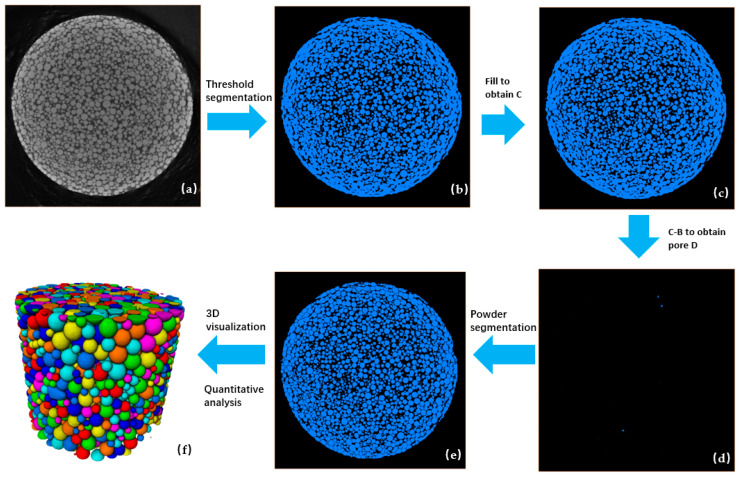
Analysis process workflow of powder CT images using Avizo 2023.1 software: (**a**) 2D CT image of the powder sample; (**b**–**e**) example of the sample state after each process step in the Avizo 2023.1 software interface; (**f**) 3D visualization of powder sample (The size, morphology etc. of particles can be presented by different colors, which can be customized).

The ROI data were imported into Avizo 2023.1 and processed using the foam analysis module and watershed algorithm for further analysis. The number of particles in each ROI is about 70,000. The minimum diameter of a particle that can be observed under the spatial resolution is about 10 μm. In this dimension of ROI, we can achieve sufficient magnification and a plentiful amount of powder numbers for analysis to ensure the accuracy of the analysis results. The analysis process workflow is illustrated in Figure 5. Import the raw data of CT images (Figure 5a) into the software and firstly use the “Interactive Thresholding” command to finish the threshold segmentation of the image, and the background brightness contrast and the light intensity are usually adjusted to allow for a clear observation of the powder particles and the hollow particle (Figure 5b). Secondly, use the “Grayscale Fill Holes” command to fill the hollow part inside the hollow particle (Figure 5c). Thirdly, extract the hollow volume (Figure 5d) in the powder sample by subtracting the analysis results of the first two steps. Fourthly, use the “Separate Object” command to separate the particles in the sample (Figure 5e). After doing these, the processed data and image can be used for analysis and 3D visualization (Figure 5f).

## 4. Quantitative Analysis of Powder Physical Properties

### 4.1. Morphology

Some researchers have analyzed the morphology of metal particles using microscopy and image analysis [19,20,21,22]. However, it is important to note that the microscopy method can only provide pseudo-3D images, rather than true 3D images, which limits the possibility of quantitative analysis. Figure 4 and Figure 5f provided a clear representation of the three-dimensional morphology of each particle. Typically, the morphology of powders was characterized using the sphericity, aspect ratio, and other similar measures. In this case, we calculated the sphericity of a single particle using Equation (1) and then determined the sphericity of the entire powder batch.

(1) $$O_{i}=\frac{4\pi {\left(\frac{3V_{p}}{4\pi }\right)}^{\frac{2}{3}}}{s_{p}}$$

(2) $$O_{\mathrm{ROI}}=\frac{\sum_{i=1}^{n}O_{i}}{n}$$

O_i_: sphericity of a single particle
V_p_: volume of a single particle
S_p_: surface area of a single particle
O_ROI_: sphericity of all the particles in the ROI

**Figure 6:**
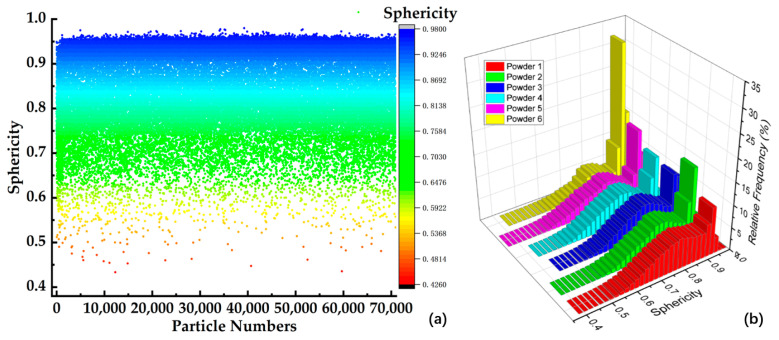
The sphericity value distribution of six samples: (**a**) the distribution of single-particle sphericity in sample six, (**b**) the sphericity distribution comparison of six samples.

The relative information of the powder can be obtained using Avizo. This included the equivalent diameter, area, and volume of each particle. The frequency count can be used to determine the distribution of the sphericity of powders. The sphericity distribution of the six samples appeared to follow a normal distribution as illustrated in Figure 6. The highest sphericity value is concentrated around 0.93, and the average sphericity of each sample listed in Table 3 is around 0.8.

**Figure 7:**
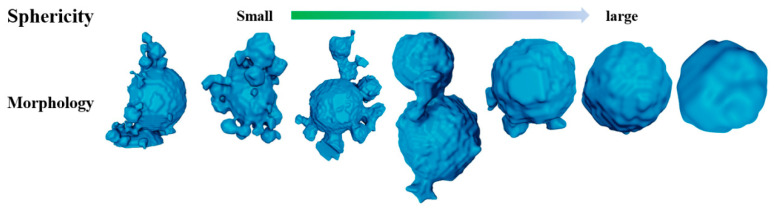
Morphology of particles with poor sphericity and the sphericity value changes with morphology.

In addition to calculating the average sphericity, it is possible to identify and examine particles with poor sphericity individually, as shown in Figure 7.

The irregularly shaped particles enhance the interaction between particles, and the more spherical the particles, the lower the resistance between them during flow, resulting in better fluidity. This is also the direction in which the gas atomization process needs to be optimized.

### 4.2. Particle Size Distribution (PSD)

The particle size distribution of Ti6Al4V powder is a crucial factor in determining the appropriate build process. The L-PBF process uses 15–53 μm powder in general, while the coarser powders are generally used in the Electron Beam Melting (EBM) or Direct Energy Deposition (DED) process. Particle size distribution analysis is commonly tested using the laser diffraction method [13], which can provide estimated particle size distributions based on a spherical particle assumption conveniently. However, the particles may be significantly non-spherical, as shown in Figure 7. It could potentially affect the accuracy of the results. Therefore, it is recommended to consider an alternative method to deal with poor sphericity particles.

**Figure 8:**
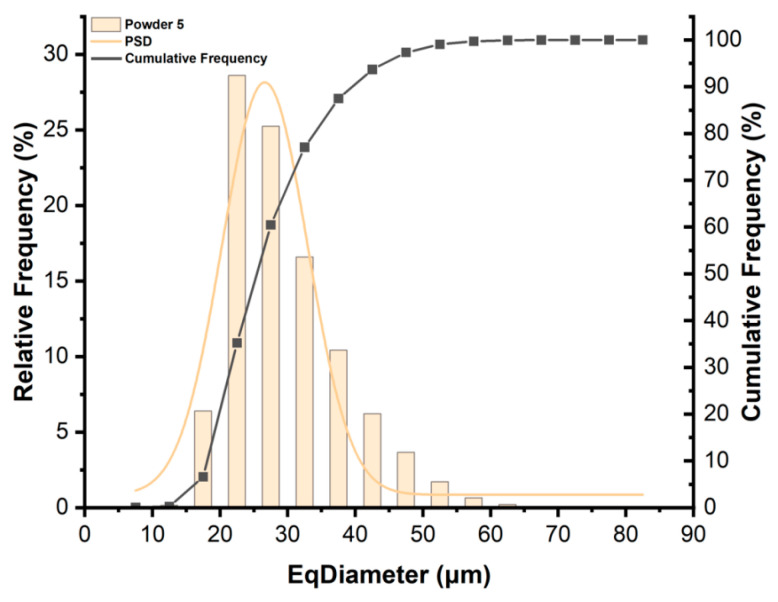
PSD curve and the cumulative frequency of Powder 5.

The eq-diameter of a single particle can be calculated using Avizo 2023.1. The PSD result of the powder sample can be obtained statistically by the frequency count of a series of particle diameter intervals in number. For example, we used the Powder 5 sample (Figure 8) to fit the PSD and obtained the D_10_, D_50_, and D_90_ values from the cumulative frequency curve.

**Figure 9:**
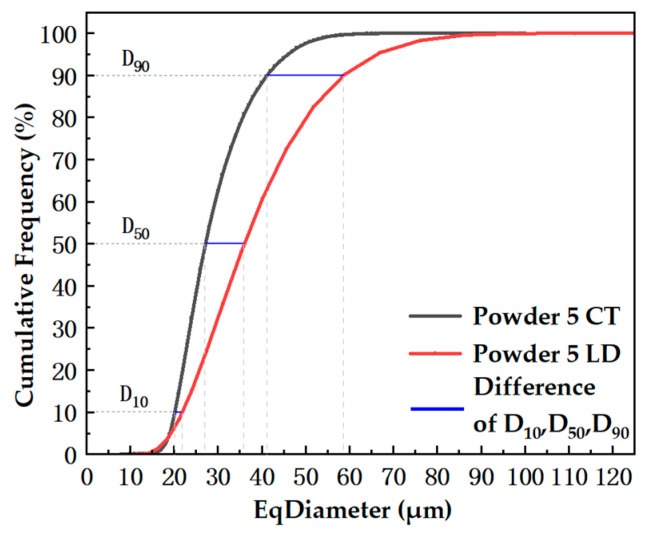
Comparison of cumulative frequency between the CT and laser diffraction method.

Figure 9 shows a comparison of the cumulative frequency between the CT and laser diffraction methods. The D_10_, D_50_, and D_90_ values of the CT method are smaller than those of the laser diffraction method. There is a difference in the principle of particle size calculation methods. The laser particle size analyzer measures particle size based on the scattering phenomenon of light. When light encounters particles (obstacles) during its journey, some of it deviates from its original propagation method, which is called scattering or diffraction of light. The smaller the particle size, the larger the scattering angle; the larger the particle size, the smaller the scattering angle. The scattering intensity also depends on the particle size and gradually decreases as the particle volume decreases. Larger particles therefore produce stronger narrow angle scattered light, while smaller particles scatter wider angles but with lower intensity. In contrast, the particle size calculated by the CT method is based on the 3D images constructed by the VGStudio Max 2023.2 and Avizo 2023.1 software which are verified by the National Institute of Standards and Technology (NIST). The CT method uses individual voxels to fill the powder particles, counts the number of voxels to obtain the entire particle volume, and then calculates the equivalent diameter. The reason we are currently considering is due to the sphericity of the particles. The better the sphericity, the closer the data between the two.

There are several standard methods to test particle size distribution nowadays, such as the sieving method, electron microscopy image analysis method, laser diffraction (scattering) method, photon coherence spectroscopy method, and particle size testing method based on particle Brownian motion, etc. The results of these methods usually have different representation forms, and cannot transfer each other. The users can compare the results tested in the same method. So, the PSD tested by CT is practicable and provides more choices for the users.

### 4.3. Hollow Particle Ratio

The hollow particle is the gas or internal porosity sealed within the particle. Due to the GA process used and technical limitations, liquid-state metal may contain gas during the solidification process which results in hollow particles. A comparison of the distributions suggested that smaller pores (<15 μm) in the powder may transfer to the as-built parts, while larger pores did not [23].

**Figure 10:**
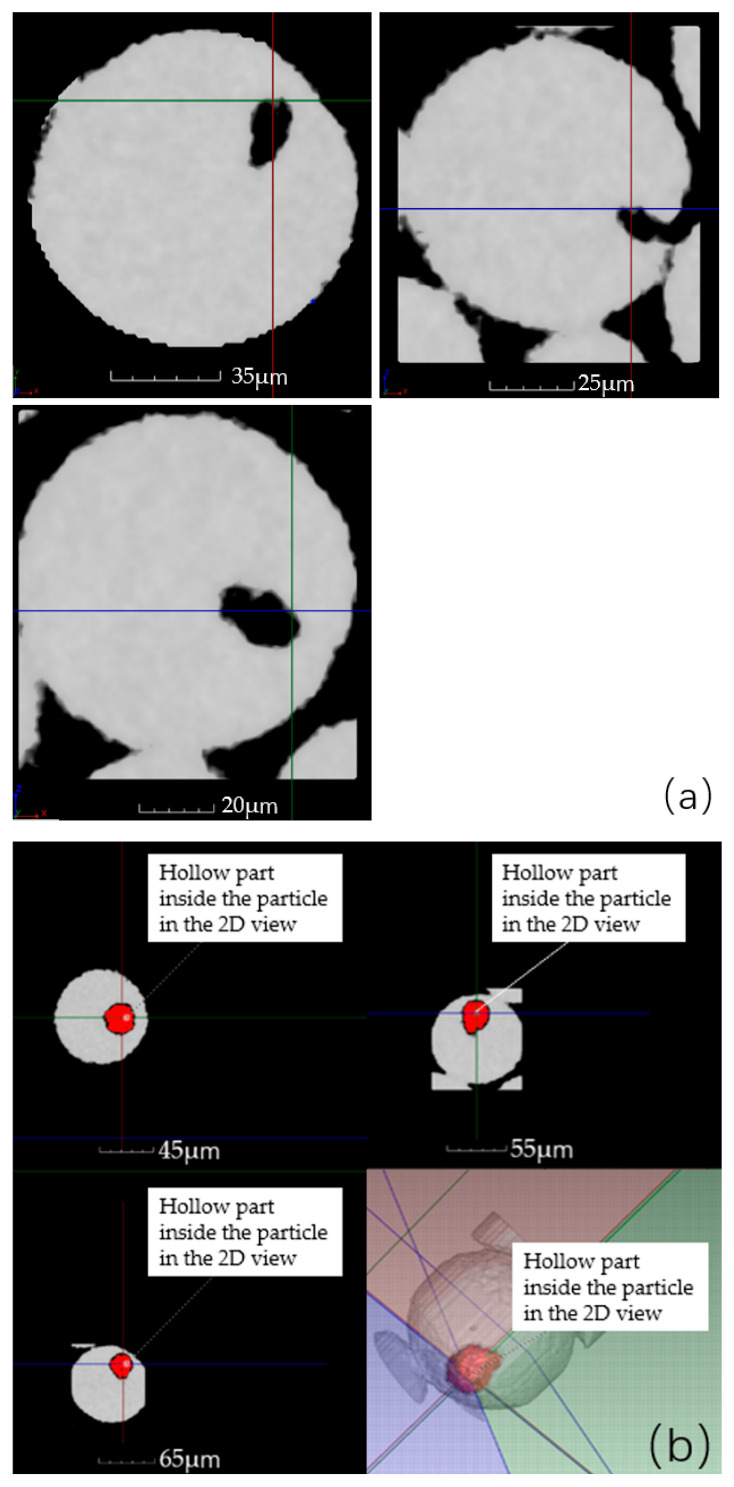
The different side views of a particle with internal porosity: (**a**) the particle with a concavity near the surface may be mistaken as a hollow particle, (**b**) locate the doubted particle and observe it from different side views to confirm whether it is a hollow particle.

Internal porosity can be visualized by sectioning particles embedded in resin using a microscope. However, this method has many disadvantages and limitations. Firstly, it is very time consuming. Secondly, the sectioning process can smear over small pores which affects the images obtained. Thirdly, the number of particles in the microscope field is small and the result is random, which needed much sectioning to obtain a statistically accurate value to avoid it. Fourthly, only the hollow particle ratio can be measured, as the morphology and dimension of the internal porosity cannot be determined through sectioning as the limited cross-section of the powders. Finally, also most importantly, it may be difficult to determine whether the observed pores are located inside the particle or simply a concavity near the surface, as depicted in the left image of Figure 10.

These disadvantages can be solved easily with micro-CT technology. CT scan data can contain 70,000 particles with a spatial resolution of 1.158 μm. Compared to the sectioning method, micro-CT can address the issue of sample scarcity. In case of doubt regarding the hollowness of a particle, it can be located in the software and viewed from other sides for confirmation, as demonstrated in Figure 10b. This is not possible with the sectioning method.

**Figure 11:**
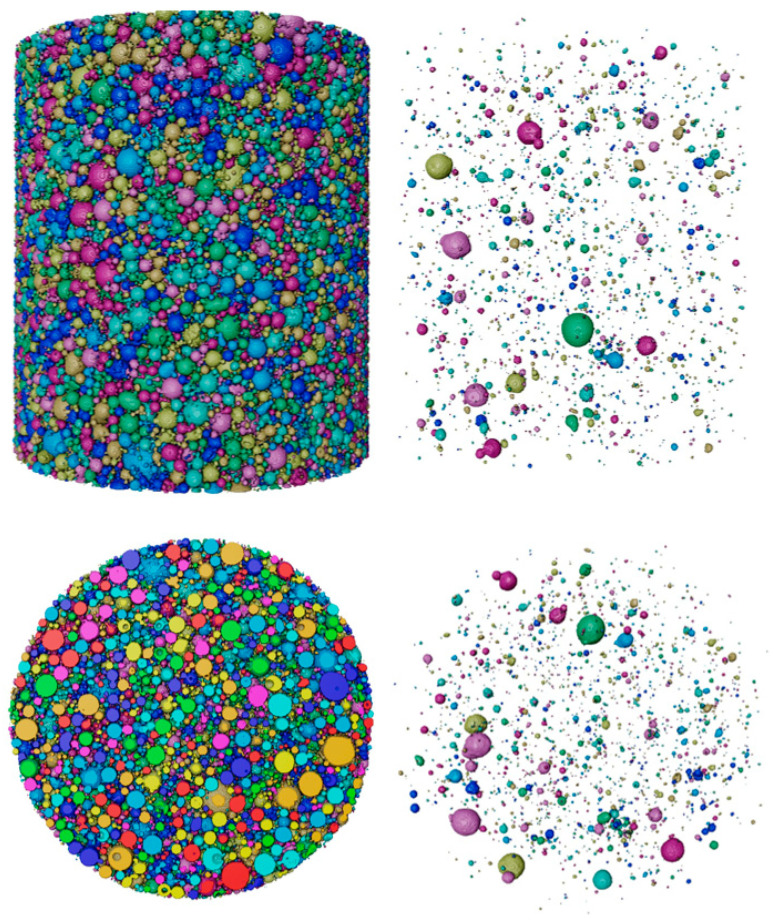
3D view of Powder 10 and 3D morphology of their internal porosities. (The size, morphology etc. of particles/hollow parts can be presented by different colors, which can be customized).

**Figure 12:**
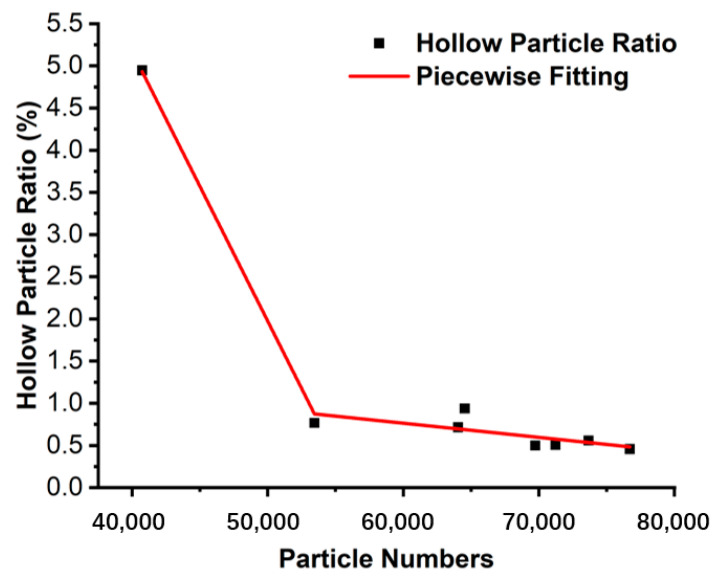
Piecewise fitting of hollow particle ratio with particle number.

Using the foam analysis module and the watershed algorithm, the 3D view of the powder is shown in Figure 11. The result showed the morphology of the internal pores, the statistical result of the number of hollow particles, and their eq-diameter, volume. The hollow particle ratio was expressed by dividing the number of hollow particles by the total number of particles in the ROI. The calculated hollow particle ratios are shown in Table 4. Figure 12 illustrates the trend using piecewise fitting of the number of particles to the hollow particle. The higher the number of particles in the same ROI volume, the finer the particles. The average diameter of Powder 10 is coarser, the number of particles is about 30,000 lower than in other samples, and we can see that the hollow particle ratio is obviously higher than in other samples. Due to the atomization mechanism and technology, the likelihood of the formation of hollow particles was greater with the increase in particle size. The fitting result is consistent with this trend.

### 4.4. Density

There are four types of density tested in the feedstock of the PBF process—the true density, the apparent density (mass per unit volume of a powder obtained following specific methods), the tapped density (mass per unit volume of a powder in a container that has been tapped under specified conditions), and the bulk density (mass per unit volume of a powder under nonstandard conditions), and the definition of these densities can be found in EN ISO 3252 [24]. The true density can be used as a preliminary evaluation index to assess whether the powder is well produced and whether there are gases or impurities in the particles. The apparent density and tapped density are more important indices in PBF. If the value of either density is lower than the requirement of the process parameters, then the density and uniformity of the powder bed layer waiting to be melted is poor, which results in porosity, lack of fusion, or other defects in the printed parts.

#### 4.4.1. True Density

**Figure 13:**
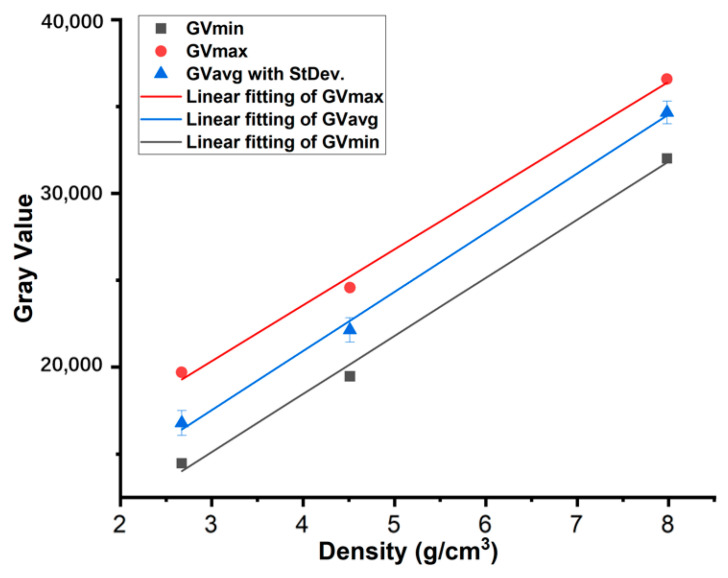
The relationship between the gray value on CT images and the true density of four powders: linear fitting of gray value and true density below 8 g/cm^3^.

The signal intensity received by the detector was related to the true density of the material tested. The four most commonly used powders in the PBF process, AlSi10Mg, Ti6Al4V, 316L, and GH4169, were mixed and scanned by micro-CT using the same parameters listed in Table 2. VGstudioMAX 2023.2 software was used to read the grayscale values in the CT images of four powders, as shown in Table 5. The gray value appeared to be linear with the density when the true density of the powder was below about 8 g/cm^3^, as shown in Figure 13.

The fitting equation for the average gray value and true density was:

y = 3405.23 × x + 7318.05(3)

y: average gray value; x: true density.

#### 4.4.2. Bulk Density

Typically, the apparent density or tapped density was commonly used to characterize the density of the powder bed. As there was no vibration in the AM process, the real density of the powder bed was uncertain. So, we need to develop a special method to test the density. Here, we calculated the volume of each particle (V_i_) in the sample holder, and then statistically calculated the whole volume of all the particles (V_p_). The V_p_ minus the void volume inside the particles V_h_ and then divided by the ROI volume (V_ROI_) and multiplied by the true density of Ti6Al4V is the bulk density (ρ_b_) of the powder sample.

The true density of Ti6Al4V powder was 4.51 g/cm^3^, while the volume of the ROI was π × 0.713^2^ × 1.602 mm^3^, and the bulk density can be calculated using Equation (4).

(4) $$\rho b_{\;}=\frac{4.51\times \left(\sum_{i=1}^{n}V_{i}\right.-\left.\sum_{i=1}^{m}V_{h}\right)}{V_{ROI}}\;g/{cm}^{3}$$

**Figure 14:**
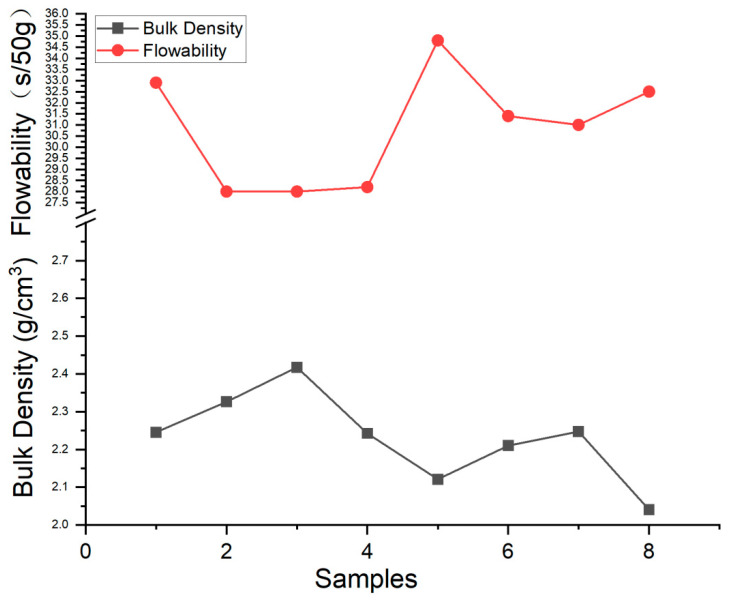
The bulk density compared with the flowability of the eight samples.

The bulk density, apparent density, and flowability of eight samples were calculated and listed in Table 6. The apparent density of Ti6Al4V particles was tested by the funnel method according to ISO 3923-1:2018 [25]. The flowability was tested according to ISO 4490:2018 [26]. From the testing results, it can be found that the bulk density values were lower than their apparent density. It was mainly caused by the fact that the sample container was fine, and the flowability affected the powder accumulation. The values of bulk density and flowability were compared in Figure 14. It was quite obvious to see that the trend of bulk density and flowability had an opposite trend.

### 4.5. Inclusion

The gray value of inclusions was different from that of the powders and can be easily recognized. Using Avizo 2023.1 software, the inclusions can be picked out and the number of inclusions can be statistically counted. The number of inclusions divided by the total number of particles was the inclusion ratio.

**Figure 15:**
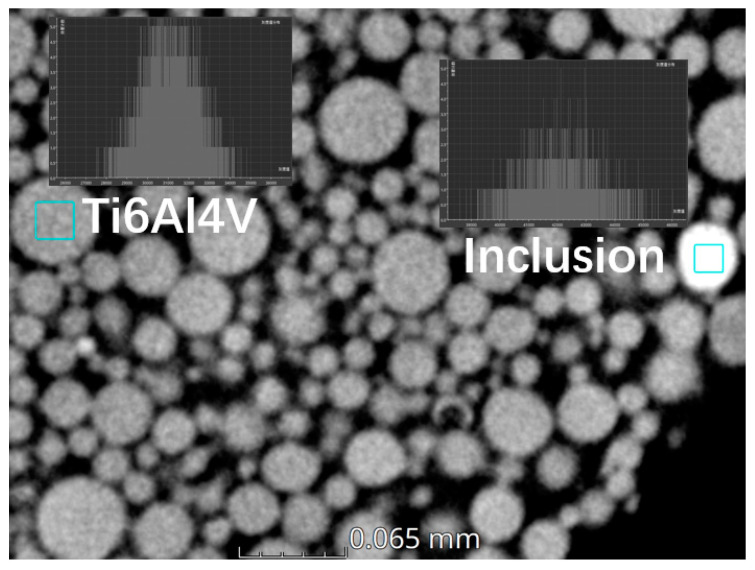
The gray value of Ti6Al4V particles and inclusions in the CT image.

Since the density of powders can be calculated, the density of the inclusions also can be obtained under a certain density value. The density of the inclusions in the powders shown in Figure 4 is calculated based on the fitting result approximately. The average gray value of inclusions (Figure 15) is 29,961.204 and the true density of inclusion is 6.65 g/cm^3^, which was calculated using the above-mentioned equations.

In addition, the morphology of inclusions can be characterized. The source of the inclusions can be inferred if the shape, density, and number of inclusions are known. As a result, there will be an improvement in powder quality for powder manufacturers.

### 4.6. Specific Surface Area

The specific surface area can affect the stock requirements of powders; the greater the specific surface area, the greater the ability to adsorb moisture. The surface area of all the particles divided by their mass is the specific area, and the calculated equation was given as follows.

(5) $$a_{s}=\frac{\sum_{i=1}^{n}s_{p}}{4.51\times \left({\sum_{i=1}^{n}V}_{p}-\sum_{i=1}^{m}V_{h}\right)}\;m^{2}/g$$

a_s_: specific surface area of powder sample
s_p_: surface area of a single particle
V_p_: volume of a single particle
V_h_: volume of hollow part inside a single particle

The specific surface area of 6 samples is show in Table 7.

The particle numbers and sphericity indeed affect the specific surface area of powders. However, the relationship of the specific surface area with particle numbers or sphericity may not be quantifiable using an equation. The use of polynomial nonlinear fitting and linear fitting tools can more clearly show the trend of their relationship.

**Figure 16:**
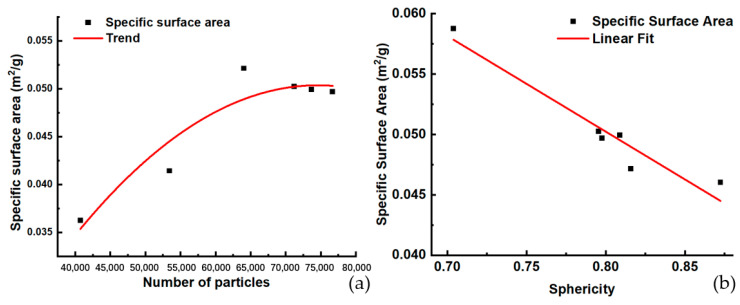
The relationship of specific surface area, particle number, and sphericity: (**a**) the trend of specific surface area with particle numbers, (**b**) the trend of specific surface area with sphericity.

Obviously, the greater the number of particles in the same volume, the smaller the particle size and the higher the specific surface area. The fitting curve shown in Figure 16a fitted this trend well.

Comparing the specific surface area of the powders with the number of particles around 70,000, the trend shown in Figure 16b showed that the higher the specific surface area, the lower the sphericity.

### 4.7. Flowability

In the PBF process, flowability had a significant impact on the uniformity of the powder bed which had further influenced the quality of the deposited powder layer. However, the significance of the flowability for powder bed layer quality was still not clear.

Flowability is a characteristic dependent on the particle ensemble’s physical properties, testing equipment, conditions, and so on [27]. Multiple testing methods can help to assess flowability, but it is not always clear which may better represent specific flow conditions or how different metrics correlate [28]. One of the most commonly used methods is the calibrated funnel method (Hall flowmeter), as described in ISO 4490. Other methods such as the Hausner ratio, Carr index, Gustavsson flowmeters, and FT4 powder rheometer can also be used, but the results of these methods were not comparable.

From the previous chapters, the other physical properties of the powders can be analyzed quantitatively using the micro-CT method. But the flowability is a dynamic property which is quite difficult to characterize through CT directly. Flowability may be related to other properties, such as morphology, particle size distribution, and true density. In this section, we tried to analyze the relationship between flowability and other physical properties of the powder such as particle morphology, particle size distribution, and so on, and these test data are shown in Table 8. The D_10_, D_50_, and D_90_ are obtained through the laser diffraction method because the CT method is not a standard method and needs more work to verify, as discussed in Section 4.2.

**Figure 17:**
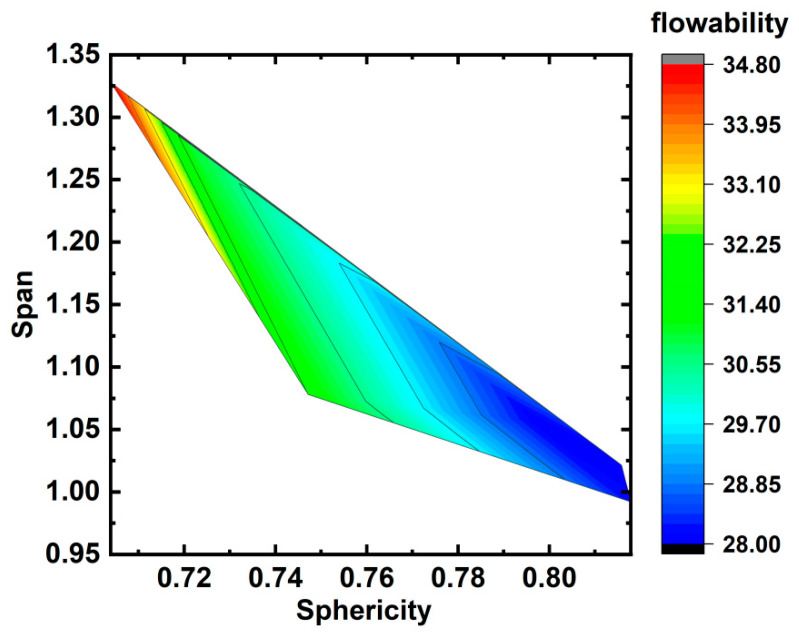
The trend of flowability with sphericity and span ((D_90_ − D_10_)/D_50_).

As the particle size distribution follows the Gaussian curve, it is not practical to analyze directly. Instead, the span, which was calculated using (D_90_ − D_10_)/D_50_, was applied to indicate the range of the particle size distribution. When the powder contained a higher concentration of fine particles, the D_10_ value decreased while the span value increased. The contour figure shown in Figure 17 was plotted using the flowability, span, and sphericity data. It can be seen that the flowability was proportional to sphericity, which was mainly caused by the fact that the particles with high sphericity experience less resistance during flow. The flowability was inversely proportional to the span at the same level of sphericity. Finer particles fill the gaps between coarser particles, and a higher concentration of finer particles increases the interaction force between powder particles, impeding the flow of powders.

## 5. Conclusions

The physical properties of Ti6Al4V powder affect the spreadability of powders and the uniformity of the powder bed, which further influences the quality of built parts. The test and analysis methods specificized in ISO/ASTM 52907:2019 can help to characterize the parameters of physical properties, but it wastes quite a lot of time and effort. The signals of micro-CT can be used to quantitatively analyze the powder properties. The analysis results revealed that the micro-CT method used for the quantitative analysis of the physical properties is an effective and highly efficient way.

The micro-CT can exhibit the 3D morphology of single particles and the sphericity of powders can be calculated with the eq-diameter, volume data, and so on. It can also locate the particles with irregular shapes and show their 3D morphology, which can provide different side views which help users to identify better.

The particle size distribution of powders can be calculated with the eq-diameter of single particles, using the data processing software to statistic the PSD of powders. Its result is similar to the laser diffraction method.

The micro-CT method was considered a relatively optimal method to measure the hollow particle ratio, and it compensated for the inferiority of the sectioning method and can show the 3D morphology of internal pores in the particles, which was useful to quantitatively statistic the hollow volume of internal pores.

In the region of density study, the gray value of CT images was proportional to their true density. So, the true density of powders can be calculated by adding some materials with a known density as a reference sample, and the bulk density of powders can be calculated with the total volume of each particle and ROI.

Different materials with different densities have different gray values in the CT images, the inclusions in the powder can be easily recognized, and the true density of the inclusions can also be calculated, which was useful for deducing the type and resource of the inclusions.

The specific surface area can be calculated using the surface area data and true density and volume of all particles in the ROI. As the specific surface area was affected by the absorbency of water, we should take special care when storing powders. In addition, the specific surface area was also related to its sphericity, and the higher the specific surface area, the lower the sphericity.

Although the flowability cannot be quantitatively tested directly by micro-CT, other properties can be used for qualitative analysis of powder flowability. The testing results revealed that the flowability was proportional to sphericity. The flowability was inversely proportional to the span at the same level of sphericity.

Some items such as particle size distribution and flowability have several test methods, and the results of different methods sometimes have no correspondence and cannot mutually convert. Although the analysis result of micro-CT is somewhat different from the testing results using other methods, this article provided a new way to characterize the properties of powders, and researchers can compare the data obtained from the same test method. This method, like others, can be studied further to become a standard. However, there is still further research required.

The research results can provide new testing methods for powder production and research and development institutions, and comprehensively judge and control powder quality from multiple aspects. The CT method assists PBF users in analyzing the relationship between the quality of AM parts and powder properties. This leads to process improvement, enhanced quality levels, increased qualification rates of AM parts, and effective powder recycling to reduce usage costs. This method can also be applied in other industries that use powder for production.

## Figures and Tables

**Table 1:** Main parameters of YXLON FF35CT.

Equipment Model	YXLON FF35CT
X-ray tube	225 kV, 320 W directional beam tube
	190 kV, 64 W transmission tube
Detector	YXLON-Panel-2530
Pixel size of the detector	139 µm
Detector pixel	1792 × 2176
Focus detector distance	280–1200 mm

**Table 2:** Scan parameters of the Ti6Al4V powders.

Items	Value	Items	Value
Voltage (kV)	80	Scan mode	Cone-beam scan
Current (μA)	80	Number of projections	1800
Filter	0.1 mm copper	Magnification	120
Mode	Microfocus	Focus size	≤2 μm

**Table 3:** The particle numbers counted and their average sphericity.

Sample No	Particle Numbers	Average Sphericity
Powder 1	71,221	0.7954
Powder 2	69,727	0.8158
Powder 3	53,437	0.8179
Powder 4	76,699	0.7977
Powder 5	73,653	0.8089
Powder 6	73,974	0.8724

**Table 4:** Statistic data of hollow particle numbers, volume, hollow particle ratio of powder samples.

Sample No	Particle Numbers	Hollow Particle Numbers	Hollow Volume (μm^3^)	Hollow Particle Ratio (%)
Powder 1	71,221	361	226,584.24	0.51
Powder 2	69,727	348	189,279.57	0.50
Powder 3	53,437	410	324,594.07	0.77
Powder 4	76,699	351	203,766.85	0.46
Powder 5	73,653	412	249,211.34	0.56
Powder 6	69,153	684	421,258.06	0.99
Powder 7	64,036	459	670,451.39	0.72
Powder 8	64,522	606	369,097.59	0.94
Powder 9	73,974	998	1,202,996.05	1.35
Powder 10	40,732	2015	8,686,273.04	4.95

**Table 5:** Gray values in CT images of four PBF powders.

Materials	True Density (g/cm^3^)	Grayscale Value			
		Min	Max	Average	StDev.
AlSi10Mg	2.67	14,470	19,709	16,783.33	716.508
Ti6Al4V	4.51	19,473	24,575	22,140.01	693.819
316L	7.98	32,010	36,583	34,655.16	650.872
GH4169	8.19	37,276	41,755	39,489.45	751.733

**Table 6:** The bulk density, apparent density, and flowability of eight Ti6Al4V powder samples.

Sample No	V_p_ (mm^3^)	ΣV_h_ (mm^3^)	V_ROI_ (mm^3^)	Bulk Density (g/cm^3^)	Apparent Density (g/cm^3^)	Flowability s/50 g
Powder 1	1.27	2.27 × 10^−4^	2.56	2.25	2.43	32.9
Powder 2	1.32	1.89 × 10^−4^	2.56	2.33	2.67	28
Powder 3	1.37	2.04 × 10^−4^	2.56	2.43	2.68	28.2
Powder 4	1.27	2.49 × 10^−4^	2.56	2.24	2.67	28
Powder 5	1.20	4.21 × 10^−4^	2.56	2.12	2.68	28.2
Powder 6	1.25	6.70 × 10^−4^	2.56	2.21	2.5	34.8
Powder 7	1.28	3.69 × 10^−4^	2.56	2.25	2.52	31.4
Powder 8	1.16	1.20 × 10^−3^	2.56	2.04	2.5	31

**Table 7:** Specific surface area of six powder samples.

Sample No.	Volume of Powders (µm^3^)	Volume of Hollow (µm^3^)	Number of Particles	Area of Powders (µm^2^)	Specific Surface Area (m^2^/g)
Powder 1	1.27 × 10^9^	2.27 × 10^5^	71,221	2.89 × 10^8^	0.0503
Powder 2	1.60 × 10^9^	3.25 × 10^5^	53,437	2.99 × 10^8^	0.0414
Powder 3	1.37 × 10^9^	2.04 × 10^5^	76,699	3.07 × 10^8^	0.0497
Powder 4	1.27 × 10^9^	2.49 × 10^5^	73,653	2.86 × 10^8^	0.0499
Powder 5	1.25 × 10^9^	6.70 × 10^5^	64,036	2.95 × 10^8^	0.0521
Powder 10	1.30 × 10^9^	8.69 × 10^5^	40,732	2.12 × 10^8^	0.0363

**Table 8:** The sphericity, PSD, and flowability data of 9 samples.

Sample No.	Sphericity	D_10_ µm	D_50_ µm	D_90_ µm	Flowability s/50 g
Powder 1	0.7954	22.5	36.5	57.3	32.9
Powder 2	0.8158	22.2	37.3	60.3	28
Powder 3	0.8179	22.4	37.2	59.3	28.2
Powder 4	0.7977	21.7	37.3	61.1	28
Powder 5	0.8089	21.9	36	58.5	28.2
Powder 6	0.7039	15.3	30.2	55.4	34.8
Powder 7	0.7472	20.1	35.9	58.8	31.4
Powder 8	0.7204	16.4	31	56.1	31
Powder 9	0.8724	23.7	36.7	56.7	32.5

## Data Availability

Data are contained within the article.
